# The impact of supported telemetric monitoring in people with type 2 diabetes: study protocol for a randomised controlled trial

**DOI:** 10.1186/1745-6215-14-198

**Published:** 2013-07-06

**Authors:** Sarah Wild, Janet Hanley, Stephanie Lewis, John McKnight, Lucy McCloughan, Paul Padfield, Mary Paterson, Hilary Pinnock, Brian McKinstry

**Affiliations:** 1 Centre for Population Health Science, University of Edinburgh, Teviot Place, Edinburgh EH8 9AG, UK; 2 Edinburgh Napier University, School of Nursing, Midwifery and Social Care, Sighthill Campus, Sighthill Court, Edinburgh EH11 4BN, UK; 3 Metabolic Unit, Western General Hospital, NHS Lothian, Crewe Road S, Edinburgh EH4 TXU, UK; 4 College of Medicine & Veterinary Medicine, The University of Edinburgh, The Queen's Medical Research Institute, 47 Little France Crescent, Edinburgh EH16 4TJ, UK; 5 Scottish Government, St Andrews House London Road, Edinburgh EH13DG, UK

**Keywords:** Diabetes, Hypertension, Glycaemic control, Blood pressure, Weight, Self-management, Telehealth

## Abstract

**Background:**

Diabetes prevalence is increasing and current methods of management are unsustainable. Effective approaches to supporting self-management are required. The aim of this randomized controlled trial is to establish whether supported telemetric monitoring of glycemic control and blood pressure results in reductions in glycosylated hemoglobin (HbA1c; the primary outcome of a measure of long-term glycemic control) and secondary outcomes of blood pressure and weight among people with poorly controlled diabetes compared to a control group receiving usual care.

**Methods/Design:**

Design: multi-center, randomized controlled trial with embedded qualitative study.

Setting: primary care in Lothian, Kent, Glasgow and Borders regions in the UK.

Participants: people with type 2 diabetes and confirmed HbA1c >7.5% (58 mmol/mol).

Intervention/comparison: randomization to intervention or control groups will be performed by the Edinburgh Clinical Trials Unit. Participants in the intervention group will be shown how to use blood glucose and blood pressure monitors and weighing scales which use Bluetooth wireless technology to transmit readings via modem to a remote server. These participants will be asked to provide at least twice weekly measurements of morning and evening blood glucose and weekly measurements of weight and blood pressure. Measurements will be checked at least weekly by practice nurses who will contact the patients to adjust therapy according to guidelines and reinforce lifestyle advice. Participants in the control group will receive usual care. All participants will receive an individual education session.

Follow-up: measurements will be performed at practices 9 months after randomization by research nurses blinded to allocation. The primary outcome measure is HbA1c and secondary outcomes measure are daytime systolic and diastolic blood pressure, weight and cost per quality-adjusted life year.

Analysis: intention-to-treat analyses will be performed. The sample size of 320 participants allows for 20% drop-out and has 80% power at 5% significance to detect a 0.5% absolute (6 mmol/mol) fall in HbA1c in the intervention group. The qualitative study will explore the experiences of patients and professionals using the intervention.

**Trial registration:**

Trial registration number ISRCTN71674628

## Background

The prevalence of diagnosed diabetes is increasing across the world and, in Scotland, has increased from 3.9% to 4.7% in people of all ages between 2006 and 2011 according to the annual Diabetes Survey [1]. Estimates of total (both diagnosed and undiagnosed) diabetes from the Association of Public Health Observatories model for people over 15 years of age in Scotland suggest that the prevalence will increase from 6.7% in 2010 to 8.6% in 2030 [2]. Annual healthcare costs of a patient with type 2 diabetes are over six times higher than the costs of a person without, largely due to the development of diabetes-related complications. However, control of blood glucose and blood pressure (as well as management of dyslipidemia) among people with diabetes reduces complications and mortality [3-5].

The current medical model for managing diabetes does not necessarily engage individuals in a self-management approach, is expensive and often not very effective partly because therapeutic inertia may result in reluctance to change treatments. Effective chronic disease management should not only reduce costs but also improve quality of care. A study in the US has found that providing healthcare professionals with current patient information improved care and reduced costs [6]. Systematic reviews indicate that engaging patients in self-monitoring and management can improve clinical outcomes in asthma [7] but the evidence that self-monitoring alone is beneficial in people with type 2 diabetes [8] is less clear. This may be because adherence to both lifestyle advice and prescribed medication for these groups is poor generally [9] but also because feedback from clinicians is often infrequent, potentially adding to patient anxiety in the presence of abnormal self-monitored readings [10].

As described previously in the protocol for a trial of telehealthcare among people with chronic obstructive pulmonary disease from our group [11], several health service policies support the need to establish the cost-effectiveness of supported self-monitoring: shifting the balance of care for people with chronic conditions to primary care [12,13]; the drive for technological solutions to healthcare problems [14]; and the importance of expert patients and self-management of long-term conditions [15,16]. While some evidence can be gleaned from international research into telemetric solutions for chronic disease management and there are encouraging results from UK and international pilot studies [17-21], further research into the cost-effectiveness of these interventions is required, particularly within the National Health Services in the UK [22]. The Telescot research program was designed using frameworks for the development and evaluation of complex interventions [23,24]. Building on existing literature [17,25,26], and in an iterative process using insights from completed and on-going exploratory and pilot work [27], we have designed several complementary Phase III randomized controlled trials. These will evaluate how telemetry-aided, supervised self-monitoring affects the management of long-term conditions in four different contexts (largely asymptomatic conditions using the example of hypertension; symptomatic, potentially unstable and progressive conditions using the example of chronic obstructive pulmonary disease; an older, more disabled group with challenging management targets using the example of hypertension among stroke survivors; co-morbid conditions using the example of diabetes, hypertension and weight management). This paper describes the protocol for the latter trial in which the aim is to establish whether supported telemetric monitoring of glycemic control and blood pressure results in reductions in glycosylated hemoglobin (HbA1c; the primary outcome of a measure of long-term glycemic control), and secondary outcomes of blood pressure and weight among people with poorly controlled diabetes compared to a control group receiving a single education session in addition to usual care.

## Methods/Design

### Design/setting/participants/ethical approval

The Telescot diabetes trial is a randomized controlled trial of supported telemetric monitoring of glycemic control, blood pressure and weight of 9 months duration with a primary outcome of HbA1c, a measure of long-term glycemic control. Secondary outcomes include blood pressure and weight and will be measured by research nurses blinded to allocation group. The setting for the trial is primary care in Lothian, Kent and Borders in the UK. Participants will have poorly controlled type 2 diabetes (defined as HbA1c>7.5% (58 mmol/mol)), be over 17 years of age, have a mobile telephone signal available at their home and each participant will have given informed consent. People with very high blood pressure (>210/135 mmHg), who have hypertension or renal disease managed in secondary care, have had treatment for a cardiac event, or other life-threatening illness within the past 6 months or have had major surgery within the last 3 months, are unable to use self-monitoring equipment, have atrial fibrillation that has not been successfully treated or are pregnant will be excluded. Approval of the trial, which will be conducted to comply with the Declaration of Helsinki, has been obtained from the South East Scotland ethics committee 2 (reference number: 10/S1102160).

### Recruitment

We will recruit socially diverse practices to the trial using both the Scottish and English Primary Care and Diabetes Research Networks. We will make use of the network of practices in Lothian we have established during a previous blood pressure trial, who are familiar with the technology and have already expressed a willingness to take part. We will also use the networks and contacts provided by our grantholders to extend recruitment beyond Lothian to Kent and Borders. Training for the practices will be provided by the research team.

Diabetes registers in participating practices will be searched to identify potential participants with HbA1c>7.5% (58 mmol/mol). Potentially eligible patients will be sent the study materials by their general practice with a covering letter and asked to contact the researcher if they are interested in participating. Once a patient has indicated interest in participating they will be invited to a clinic in their general practitioner’s (GP’s) surgery run by a research nurse. At the initial appointment the nurse will go through the study information with the patient, take written informed consent (which will cover consent to have their eligibility checked as well as their consent to take part in the trial), check potential eligibility, ask the patient to complete a short questionnaire on their demographic characteristics, past and current self-monitoring of blood pressure and blood glucose, collect a blood sample for HbA1c and cholesterol measurement, a urine sample for sodium:creatinine ratio and initiate ambulatory blood pressure monitoring using the Spacelab Ambulatory Blood Pressure Monitor (ABPM) [28]. The nurse will also explain the process for recruitment and potential randomization if patients are eligible.

All patients will be loaned an ABPM with which they can monitor their blood pressure for 14 hours. However, if they wish they can delay collecting ABPM data until after the result of their HbA1C is available and their eligibility is confirmed. Once the HbA1c result is available, any ineligible patients will be telephoned to let them know that they do not need to return for a second appointment to gather baseline measurements. Patients’ ABPM results will be released to the practice.

### Baseline measurements

At their second appointment, prior to randomization, participants will have the following baseline measurements taken: smoking history, height and weight, and exhaled carbon monoxide. In addition they will be asked to complete a range of questionnaires to collect information on anxiety/depression [29], quality of life [30], self-efficacy [31], medication adherence [32], physical activity [33], exercise tolerance [34], knowledge of managing diabetes [35] and ethnic group, based on categories included in the 2011 Census [36].

Randomization will be carried out remotely at the Edinburgh Clinical Trials Unit to ensure adequate concealment. We will use minimization with a random element based on age (<70 or ≥70 years of age), sex, location (that is, Lothian, Kent, Glasgow or Borders) use of two or more diabetes drugs, use of three or more hypertension drugs and glucose self-monitoring history in order to make sure that these factors are distributed equally between intervention and usual care groups. The allocated treatment code will be generated by the Edinburgh Clinical Trials Unit over the internet (with a telephone back-up option if required). The participant will then be informed about the arm of the trial to which they have been assigned and given appropriate information and demonstration of equipment if allocated to the intervention group. All randomized participants will be included in the intention-to-treat analysis. All participants, regardless of allocation group, will be seen by a healthcare professional from the practice trained in the management of type 2 diabetes who will assess current control, optimize management in line with Scottish Intercollegiate Guidelines Network and National Institute of Health and Clinical Excellence (NICE) guidelines and deliver a one-to-one standardized education session.

It is not possible to blind clinicians or patients to the allocation group but baseline data collection will be undertaken by the research nurses before randomization takes place. Patients will be requested not to reveal their allocation to research nurses undertaking follow-up measurements, although we recognize that inadvertent references by the patients or in their primary care record may reveal their allocation. The use of objective outcomes (HbA1c, ambulatory blood pressure, weight, validated questionnaires) will also reduce the possibility of bias. Patients will be asked not to reveal which group they are in or to return their equipment until the final monitoring has been carried out. The research nurses will be asked to record unblinding prior to the final assessment.

### Intervention

Patients in the intervention group will be given blood pressure and blood glucose monitors and weighing scales which use Bluetooth to transmit readings via a (supplied) modem to a remote server. The user may securely access their record on the server at any time (either at home if they have internet access, or in a library or other public internet access point). Their GP and practice nurse will also be able to access this record via the Internet. Users will also receive regular (monthly) feedback based on their blood pressure and blood glucose over the past 10 readings which will be sent by post or email according to the patient’s wishes.

Participants will be trained in use of the equipment and the demonstration will be backed up by written information. The written information pack will also contain leaflets about initiating or maintaining lifestyle changes and how long it is likely to be before any lifestyle or medication change will have an impact on their rolling average blood pressure or blood glucose. The suggested lifestyle changes will include weight control, reducing salt in the diet and increasing physical activity.

The user will also be given contact details for the practice nurse and encouraged to make contact by email or by telephoning at specified times if their blood pressure or blood glucose remains high, to discuss their treatment. In the event of a very high blood pressure (>220 mmHg) or very high blood glucose levels (>15 mmol/l) patients will be asked to repeat the measurements and, if they remain high, to contact their practice urgently for advice. Patients recording very low blood glucose (<4 mmol/l) will be advised to eat or take a glucose drink and, if hypoglycemia persists, to contact the practice. Practice nurses will be encouraged to review the readings they are sent about patients whose blood pressure or blood glucose remains high or persistently low or who have not checked their blood pressure or blood glucose within the agreed timeframe on a weekly basis. Practice nurses may choose to contact the patient to offer further treatment or support and will be asked to record such contacts in the normal practice electronic record. The nurses will also be asked to transfer the current average blood pressure and blood glucose into the primary care record.

Patients will be encouraged to check their blood pressure 10 to 20 times over the first seven days to establish a reliable average and then about 4 times a month if their average blood pressure is at or below the target of 130 mmHg. If blood pressure is above the target and any changes to lifestyle or medication are made, the users will be asked to undertake another more intensive period of monitoring (10 to 20 readings over seven days) after an appropriate time (usually 3 to 4 weeks depending on medication change) in order to establish the impact on the rolling average blood pressure. The guidance will not be restrictive as this tool is being provided for patients to use in order to understand their blood pressure better and to better manage their own health.

Patients in the intervention group will be asked to provide at least twice weekly measurements of morning and evening blood glucose and weekly measurements of morning weight. These measurements will be automatically forwarded to the study website and checked at least weekly by the practice nurse. Patients with high readings will be highlighted by the software for easy identification. The nurses will contact the patients by telephone, text or email to adjust therapy and reinforce lifestyle advice on the need for more or less intensive monitoring. Antihypertensive drug treatment may be altered every 4 to 6 weeks until mean blood pressure based on the last 10 readings is at or below the target of 130 mmHg. Treatment for diabetes will be altered according to the protocol based on guidelines every 4 weeks with the aim of maintaining consecutive fasting blood levels between 4 and 6 mmol/l. Patients who require the addition of insulin to their treatment during the course of the trial will be jointly managed by the practice nurse and GP along with specialist diabetes nurses at the local hospital (if appropriate) according to local practice, all of whom will have access to the telemetric data for patients in the intervention group. Algorithms for glucose and blood pressure management are given in the appendices.

### Control group

Patients in the control group will receive leaflets with lifestyle advice and usual care, that is attending the practice nurse or GP for appointments as normal. They will be asked about their use of self-monitoring of blood pressure and blood glucose at baseline and follow-up. Patients with uncontrolled blood pressure in our recent audit saw the nurse or doctor three or four times on average in 6 months and we would anticipate a similar number of attendances for patients with both uncontrolled glycemia and blood pressure. Patients requiring insulin during the course of the trial will be managed jointly as above with specialist diabetes nurse support. Throughout the trial, patients will be reviewed according to clinical need by their normal clinical advisors. Clinical care in both groups for patients in Lothian will be in accordance with NHS Lothian protocols which are based on the recommendations of national and international guidelines, and in other areas according to NICE guidance. Therapeutic algorithms for both controls and intervention will follow guidelines, based upon the NICE guidelines [37] using a standard stepped treatment program for blood glucose.

### Follow-up measurements

Participants will be seen by a researcher/research nurse blinded to their allocation at their GP practice 9 months after randomization. The follow-up appointment will include completion of questionnaires to collect information as at baseline, other than ethnicity, blood sample collection for HbA1c and cholesterol and fitting of the ABPM. Participants will be asked to return the ABPM the following day. Use of healthcare resources (number and duration of hospital admissions, including whether primary diagnosis was diabetes-related, practice and out-of-hours consultations, routine reviews for blood pressure or diabetes, prescriptions for antihypertensive and diabetes drugs and test-strips) and adverse events will be extracted from the participant’s electronic record by the researcher or research nurse at the follow-up appointment. Electronic records will be retrieved and analyzed for compliance with monitoring. The baseline measures (excluding height) will be repeated at the end of the 9 month follow-up period.

### Data collection and statistical analysis

Records will be kept of the numbers of practices and patients that were approached to participate in the study and the numbers and proportions of practices and patients that consented or declined to take part. Data from participating individuals will be entered from questionnaires and paper records by the researcher to a database. A random 10% sample will be checked for accuracy by independent trial staff. If we detect systematic errors we will re-enter all the data. Every attempt will be made to collect complete data on all of the participants.

Data analysis will be carried out by the Edinburgh Clinical Trials Unit. Data will be analyzed on an intention-to-treat basis, that is by allocation group regardless of compliance with the intervention. In the primary analysis we will remove patients with missing outcome data, but will perform sensitivity analyses to investigate the effect of this. Binary outcomes will be presented both as relative and absolute risk reductions. For HbA1c and blood pressure, and other normally distributed measurements that are taken at baseline and follow-up, adjustment for baseline measures will be performed using analysis of covariance. For the primary outcome, subgroup analyses will be performed based on sex, tertiles of age and socio-economic status (based on the Scottish Index of Multiple Deprivation derived from postcode) and tertiles of baseline HbA1c, home self-monitored systolic blood pressure and body mass index. These factors may all reasonably be hypothesized to influence the effect of the intervention. Subgroup analyses will be performed by adding any interaction between these factors and treatment into the analysis of covariance model and observing whether the fit of the model is statistically significantly improved. No interim analysis is planned.

### Sample size calculation

There were 4440 people with type 2 diabetes, an HbA1c of >7.5% and systolic blood pressure of ≥135 mmHg on the Lothian diabetes register in 2008. This group reflects approximately 16% of people on the register with type 2 diabetes. Their mean (standard deviation) HbA1c was 8.8% (1.4%) and their mean (standard deviation) systolic blood pressure was 149 mmHg (13 mmHg).

Using these values, we calculate that a randomized controlled trial with 125 people completing each arm with no change in values in the control group at the end of the trial would have 80% power at 5% significance to detect a 0.5% absolute fall in HbA1c and a 5 mmHg fall in systolic blood pressure in the intervention group. In an earlier trial of telemetric monitoring of blood pressure, 60% of patients on a practice hypertension register agreed to take part and 82% were retained within the trial [38]; we anticipate similar recruitment and retention rates in this study. We therefore plan to recruit a total of approximately 320 patients to ensure two final group sizes of 125 people which will provide sufficient power (assuming that up to 20% of people may drop out of the trial). We anticipate that this will involve recruiting 25 to 30 large general practices to participate in the study.

### Health economic analysis

The health economic analyses will assess the cost-effectiveness of the tele-supported self-monitoring compared to usual care. A cost-utility analysis (incremental cost per quality-adjusted life year) will be performed. The perspective will be the NHS. The benefits will include health outcomes measured in terms of quality-adjusted life years which will be derived from the responses to the EQ-5D [30]. Health service (GP/nurse consultations, telephone consultations, home visits, accident and emergency attendances, out-patient consultations, hospitalizations) and drug use over the trial period will be extracted from practice records to check the validity of self-reporting. Nurses will be asked how much time they use checking the website and on the telephone to patients. The costs of the tele-monitoring equipment and the set-up and support costs will be estimated using expert judgment. Resource use estimates will be combined with unit costs obtained from standard sources such as the Personal Social Services Research Unit. The results of the economic evaluation will be presented as an incremental cost-effectiveness ratio (cost-utility analysis). The evaluation will include both deterministic and probabilistic sensitivity analysis. Modeling of longer term costs and benefits will be explored using estimates from published studies including UKPDS [39]. Advice on the health economics analysis is being provided by Dr Marjon van der Pol from the Health Economics Research Unit at the University of Aberdeen and Andrew Stoddart from the Health Services Research Unit at the University of Edinburgh.

### Qualitative study

#### Participants

A sub-sample of participants in the randomized controlled trial will be recruited to the nested qualitative study. Up to 20 participants from the intervention group will be recruited for semi-structured interviews plus a further 10 to 12 for two focus groups. The purpose of the focus groups will be to allow the group to explore shared ideas and experiences relating to type 2 diabetes and hypertension and their experiences in managing it. The interviews with individuals will cover similar subjects but will allow private discussion of issues such as not using medication in the prescribed way (which people may not be willing to discuss in a public situation). A maximum variation sample in relation to age, social class, ethnicity (if ethnicity varies sufficiently between participants) and severity of type 2 diabetes and hypertension, plus level of use of the system will be sought.

#### Healthcare professionals

Up to 20 professionals participating in the trial will be interviewed (face-to-face or by telephone according to the preference of the clinician). This will include the specialist diabetes nurses providing the support services, long-term condition nurses, and representatives of primary and secondary care services.

#### Topic guides

The initial guide for semi-structured interviews with patients will be based on the themes identified from the literature and the on-going studies, but the guide will be reviewed and refined iteratively as data are gathered and analyzed and new themes arise. Participants will therefore be encouraged to give their views on the usefulness of the systems in general and then tell their own story about managing their own long-term conditions and the impact of the systems.

Interviews with professionals will seek to investigate perceptions of the benefits (or otherwise) of the intervention, experiences of implementing and maintaining it and the barriers and facilitators they have experienced.

#### Analysis

The interviews and focus groups will be fully transcribed. The Framework method will be used to classify and organize data according to key themes, concepts and emergent categories [40]. Data will be analyzed from the theoretical perspective of the diffusion of innovations literature and the behavior change literature, underpinned by social learning theory, which emphasize the importance of people’s perceptions in understanding their behavior in relation to an innovation [41-45]. Initial coding will be carried out by the qualitative researcher with reference to the transcripts and voice recordings and the analysis recorded using NVivo 7 QSR international Pty Ltd’ 2nd Floor, 651 Doncaster Road, Doncaster, Victoria 3108 Australia Constant comparison (checking experiences against those of others in the sample) will ensure that the thematic analysis represents all perspectives and negative cases will be sought. The analysis will then be reviewed by the wider research team to aid interpretation. Validity checking of the analysis will include recoding of some interviews by an independent researcher and coding review of some of the data by a patient reference group.

## Discussion

The proposed trial will provide additional information on the cost-effectiveness and feasibility of supported telemetric monitoring of two common, chronic conditions, diabetes and hypertension, whose prevalence is expected to continue increasing across the world. Limitations of the trial include the potential for unblinding of research nurses undertaking follow-up measurements to allocated group and the inability to identify whether the intervention may be more effective in patient sub-groups, for example people with newly diagnosed diabetes or who may shortly require insulin treatment. In addition the assumption that outcome measures among the comparison group will remain unchanged may not hold which will either strengthen or weaken the power of the study depending on the direction in which change occurs. The study design also precludes investigation of whether effectiveness of the intervention would be influenced by separate monitoring of blood pressure and glycemic control in different groups as this would require a factorial design. The standard limitation of all health services research that it is difficult to generalize the findings regarding feasibility to other settings also applies to this work, but the effectiveness of the intervention may have wider application to other populations.

## Trial status

Ongoing. Recruitment started in August 2011 and follow-up is expected to be complete by April 2014.

## Abbreviations

ABPM: Ambulatory blood pressure monitor; GP: General Practitioner; HbA1c: glycosylated hemoglobin; NICE: National Institute of Health and Clinical Excellence.

## Competing interests

The authors declare that they have no competing interests.

## Authors’ contributions

SW drafted the manuscript on the basis of the study protocol. BM and JH conceived the study. All authors participated in study design and have read and approved the final manuscript.

## References

[B1] Scottish Diabetes Survey Monitoring Group Scottish Diabetes Survey 2011 Edinburgh: Scottish Government http://www.diabetesinscotland.org.uk/Publications.aspx. 2012. 8-11-2012

[B2] Holman N Forouhi NG Goyder E Wild SH The Association of Public Health Observatories (APHO) diabetes prevalence model: estimates of total diabetes prevalence for England, 2010–2030 Diabet Med 2011 28 575 582 21480968 10.1111/j.1464-5491.2010.03216.x

[B3] Holman RR Paul SK Bethel MA Neil HA Matthews DR Long-term follow-up after tight control of blood pressure in type 2 diabetes N Engl J Med 2008 359 1565 1576 10.1056/NEJMoa0806359 18784091

[B4] Holman RR Paul SK Bethel MA Matthews DR Neil HA 10-year follow-up of intensive glucose control in type 2 diabetes N Engl J Med 2008 359 1577 1589 10.1056/NEJMoa0806470 18784090

[B5] Colhoun HM Betteridge DJ Durrington PN Hitman GA Neil HA Livingstone SJ Thomason MJ Mackness MI Charlton-Menys V Fuller JH CARDS investigators Primary prevention of cardiovascular disease with atorvastatin in type 2 diabetes in the Collaborative Atorvastatin Diabetes Study (CARDS): multicentre randomised placebo-controlled trial Lancet 2004 364 685 696 10.1016/S0140-6736(04)16895-5 15325833

[B6] Hivert MF Grant RW Shrader P Meigs JB Identifying primary care patients at risk for future diabetes and cardiovascular disease using electronic health records BMC Health Serv Res 2009 9 170 10.1186/1472-6963-9-170 19772639 PMC2753330

[B7] Gibson PG Powell H Coughlan J Wilson AJ Abramson M Haywood P Baumann A Hensley MJ Walters EH Self-management education and regular practitioner review for adults with asthma Cochrane Database Syst Rev 2003 1 CD001117 12535399 10.1002/14651858.CD001117

[B8] Farmer A Wade A Goyder E Yudkin P French D Craven A Holman R Kinmonth A-L Neil A Impact of self monitoring of blood glucose in the management of patients with non-insulin treated diabetes: open parallel group randomised trial BMJ 2007 335 132 10.1136/bmj.39247.447431.BE 17591623 PMC1925177

[B9] Messerli FH Williams B Ritz E Essential hypertension Lancet 2007 370 591 603 10.1016/S0140-6736(07)61299-9 17707755

[B10] Peel E Parry O Douglas M Lawton J Blood glucose self-monitoring in non-insulin-treated type 2 diabetes: a qualitative study of patients’ perspectives Br J Gen Pract 2004 54 183 188 15006123 PMC1314828

[B11] Pinnock H Hanley J Lewis S MacNee W Pagliari C van der Pol M Sheikh A McKinstry B TELESCOT Programme Group The impact of a telemetric chronic obstructive pulmonary disease monitoring service: randomised controlled trial with economic evaluation and nested qualitative study Prim Care Respir J 2009 18 233 235 10.4104/pcrj.2009.00040 19588056 PMC6619269

[B12] Department of Health Our health, our care, our say: a new direction for community services 2005 London: The Stationery Office

[B13] Scottish Executive Health Department A national framework for service change in the NHS in Scotland. Building a health service fit for the future 2005 Edinburgh: Scottish Executive

[B14] Department of Health Information for health: an information strategy for the modern NHS 1998–2005 1998 London: Department of Health

[B15] Department of Health The Expert Patient: A new approach to chronic disease management for the 21st century 2001 London: The Stationery Office

[B16] Department of Health Improving Chronic Disease Management 2004 London: The Stationery Office

[B17] Shea S Weinstock RS Starren J Teresi J Palmas W Field L Morin P Goland R Izquierdo RE Wolff LT Ashraf M Hilliman C Silver S Meyer S Holmes D Petkova E Capps L Lantigua RA A randomized trial comparing telemedicine case management with usual care in older, ethnically diverse, medically underserved patients with diabetes mellitus J Am Med Inform Assoc 2006 13 40 51 10.1197/jamia.M1917 16221935 PMC1380195

[B18] McMahon GT Gomes HE Hickson HS Hu TM Levine BA Conlin PR Web-based care management in patients with poorly controlled diabetes Diabetes Care 2005 28 1624 1629 10.2337/diacare.28.7.1624 15983311 PMC1262644

[B19] Marrero DG Vandagriff JL Kronz K Fineberg NS Golden MP Gray D Orr DP Wright JC Johnson NB Using telecommunication technology to manage children with diabetes: the Computer-Linked Outpatient Clinic (CLOC) study Diabetes Educ 1995 21 313 319 10.1177/014572179502100409 7621734

[B20] Casas A Troosters T Garcia-Aymerich J Roca J Hernandez C Alonso A del Pozo F de Toledo P Antó JM Rodríguez-Roisín R Decramer M members of the CHRONIC Project Integrated care prevents hospitalisations for exacerbations in COPD patients Eur Respir J 2006 28 123 130 10.1183/09031936.06.00063205 16611656

[B21] Niesink A de Weert-van Oene G Schrijvers AJ The effects of integrated care for COPD patients on quality of life. Review and research design Prim Care Resp J 2006 15 2001

[B22] Whitten PS Mair FS Haycox A May CR Williams TL Hellmich S Systematic review of cost effectiveness studies of telemedicine interventions BMJ 2002 324 1434 1437 10.1136/bmj.324.7351.1434 12065269 PMC115857

[B23] Medical Research Council A framework for the development and evaluation of RCTs for complex interventions to improve health 2000 London: Medical Research Council

[B24] Campbell NC Murray E Darbyshire J Emery J Farmer A Griffiths F Guthrie B Lester H Wilson P Kinmonth AL Designing and evaluating complex interventions to improve health care BMJ 2007 334 455 459 10.1136/bmj.39108.379965.BE 17332585 PMC1808182

[B25] de Toledo P Jimenez S del Pozo F Roca J Alonso A Hernandez C Telemedicine experience for chronic care in COPD IEEE Trans Inf Technol Biomed 2006 10 567 573 10.1109/TITB.2005.863877 16871726

[B26] Clark RA Inglis SC McAlister FA Cleland JG Stewart S Telemonitoring or structured telephone support programmes for patients with chronic heart failure: systematic review and meta-analysis BMJ 2007 334 942 950 10.1136/bmj.39156.536968.55 17426062 PMC1865411

[B27] Pinnock H Slack R Pagliari C Price D Sheikh A Understanding the potential role of mobile phone-based monitoring on asthma self-management: qualitative study Clin Exp Allergy 2007 37 794 802 10.1111/j.1365-2222.2007.02708.x 17456228

[B28] O’Brien E Mee F Atkins N O’Malley K Accuracy of the SpaceLabs 90207 determined by the British Hypertension Society protocol J Hypertens 1991 9 573 574 10.1097/00004872-199106000-00016 1653299

[B29] Zigmond AS Snaith RP The hospital anxiety and depression scale Acta Psychiatr Scand 1983 67 361 370 10.1111/j.1600-0447.1983.tb09716.x 6880820

[B30] The Measurement and Valuation of Health Status Using EQ-5D A European Perspective: Evidence from the EuroQol BIO MED Research Programme 2003 Dordrecht: Kluwer Academic Publishers

[B31] Lorig KR Sobel DS Ritter PL Laurent D Hobbs M Effect of a self-management program on patients with chronic disease Eff Clin Pract 2001 4 256 262 11769298

[B32] Morisky DE Green LW Levine DM Concurrent and predictive validity of a self-reported measure of medication adherence Med Care 1986 24 67 74 10.1097/00005650-198601000-00007 3945130

[B33] Craig CL Marshall AL Sjostrom M Bauman AE Booth ML Ainsworth BE Pratt M Ekelund U Yngve A Sallis JF Oja P International physical activity questionnaire: 12-country reliability and validity Med Sci Sports Exerc 2003 35 1381 1395 10.1249/01.MSS.0000078924.61453.FB 12900694

[B34] Myers J Bader D Madhavan R Froelicher V Validation of a specific activity questionnaire to estimate exercise tolerance in patients referred for exercise testing Am Heart J 2001 142 1041 1046 10.1067/mhj.2001.118740 11717610

[B35] Fitzgerald JT Funnell MM Hess GE Barr PA Anderson RM Hiss RG Davis WK The reliability and validity of a brief diabetes knowledge test Diabetes Care 1998 21 706 710 10.2337/diacare.21.5.706 9589228

[B36] General Register Office for Scotland Scotland’s census 2011: recommendations on content 2011 Edinburgh: General Register Office for Scotland

[B37] National Institute for Health and Clinical Excellence NICE guideline CG66 Type 2 diabetes: the management of type 2 diabetes (update) 2008 London: NICE

[B38] McKinstry B Hanley J Heaney D McCloughan L Elton R Webb DJ Impact on hypertension control of a patient-held guideline: a randomised controlled trial Br J Gen Pract 2006 56 842 847 17132351 PMC1927092

[B39] Stratton IM Adler AI Neil HA Matthews DR Manley SE Cull CA Hadden D Turner RC Holman RR Association of glycaemia with macrovascular and microvascular complications of type 2 diabetes (UKPDS 35): prospective observational study BMJ 2000 321 405 412 10.1136/bmj.321.7258.405 10938048 PMC27454

[B40] Casey D De Civita M Dasgupta K Understanding physical activity facilitators and barriers during and following a supervised exercise programme in Type 2 diabetes: a qualitative study Diabet Med 2010 27 79 84 10.1111/j.1464-5491.2009.02873.x 20121893

[B41] Rogers E Diffusion of Innovations 2003 London: Macmillan

[B42] Bandura A Social learning theory 1977 Englewood Cliffs, New Jersey: Prentice-Hall

[B43] Bandura A Social Foundations of Thought and Action – A Social Cognitive Theory 1986 Englewood Cliffs, New Jersey: Prentice-Hall

[B44] Wiliams F Gibbons DV Technology Transfer: A Communication Perspective 1990 Newbury Park: Sage

[B45] Mittman BS Tonesk X Jacobson PD Implementing clinical practice guidelines: social influence strategies and practitioner behavior change QRB Qual Rev Bull 1992 18 413 422 1287523 10.1016/s0097-5990(16)30567-x

